# Candidate target genes of the male-specific expressed *Doublesex* in the termite *Reticulitermes speratus*

**DOI:** 10.1371/journal.pone.0299900

**Published:** 2024-03-01

**Authors:** Kokuto Fujiwara, Satoshi Miyazaki, Kiyoto Maekawa

**Affiliations:** 1 Graduate School of Science and Engineering, University of Toyama, Gofuku, Toyama, Japan; 2 Graduate School of Agriculture, Tamagawa University, Machida, Tokyo, Japan; 3 Academic Assembly, University of Toyama, Gofuku, Toyama, Japan; Shanghai Ocean University, CHINA

## Abstract

Eusocial insects such as termites, ants, bees, and wasps exhibit a reproductive division of labor. The developmental regulation of reproductive organ (ovaries and testes) is crucial for distinguishing between reproductive and sterile castes. The development of reproductive organ in insects is regulated by sex-determination pathways. The sex determination gene *Doublesex* (*Dsx*), encoding transcription factors, plays an important role in this pathway. Therefore, clarifying the function of *Dsx* in the developmental regulation of sexual traits is important to understand the social evolution of eusocial insects. However, no studies have reported the function of *Dsx* in hemimetabolous eusocial group termites. In this study, we searched for binding sites and candidate target genes of *Dsx* in species with available genome information as the first step in clarifying the function of *Dsx* in termites. First, we focused on the *Reticulitermes speratus* genome and identified 101 candidate target genes of *Dsx*. Using a similar method, we obtained 112, 39, and 76 candidate *Dsx* target genes in *Reticulitermes lucifugus*, *Coptotermes formosanus*, and *Macrotermes natalensis*, respectively. Second, we compared the candidate *Dsx* target genes between species and identified 37 common genes between *R*. *speratus* and *R*. *lucifugus*. These included several genes probably involved in spermatogenesis and longevity. However, only a few common target genes were identified between *R*. *speratus* and the other two species. Finally, *Dsx* dsRNA injection resulted in the differential expression of several target genes, including *piwi-like protein* and *B-box type zinc finger protein ncl-1* in *R*. *speratus*. These results provide valuable resource data for future functional analyses of *Dsx* in termites.

## Introduction

The sex determination pathway in insects comprises genes related to the regulatory functions of splice variant formation and transcription [1–3]. It is a well-conserved pathway involved not only in sex determination during embryogenesis but also in sex-specific morphogenesis during post-embryonic development, including sexual dimorphism in flies and some scarab beetles [4–7].

**Fig 1:**
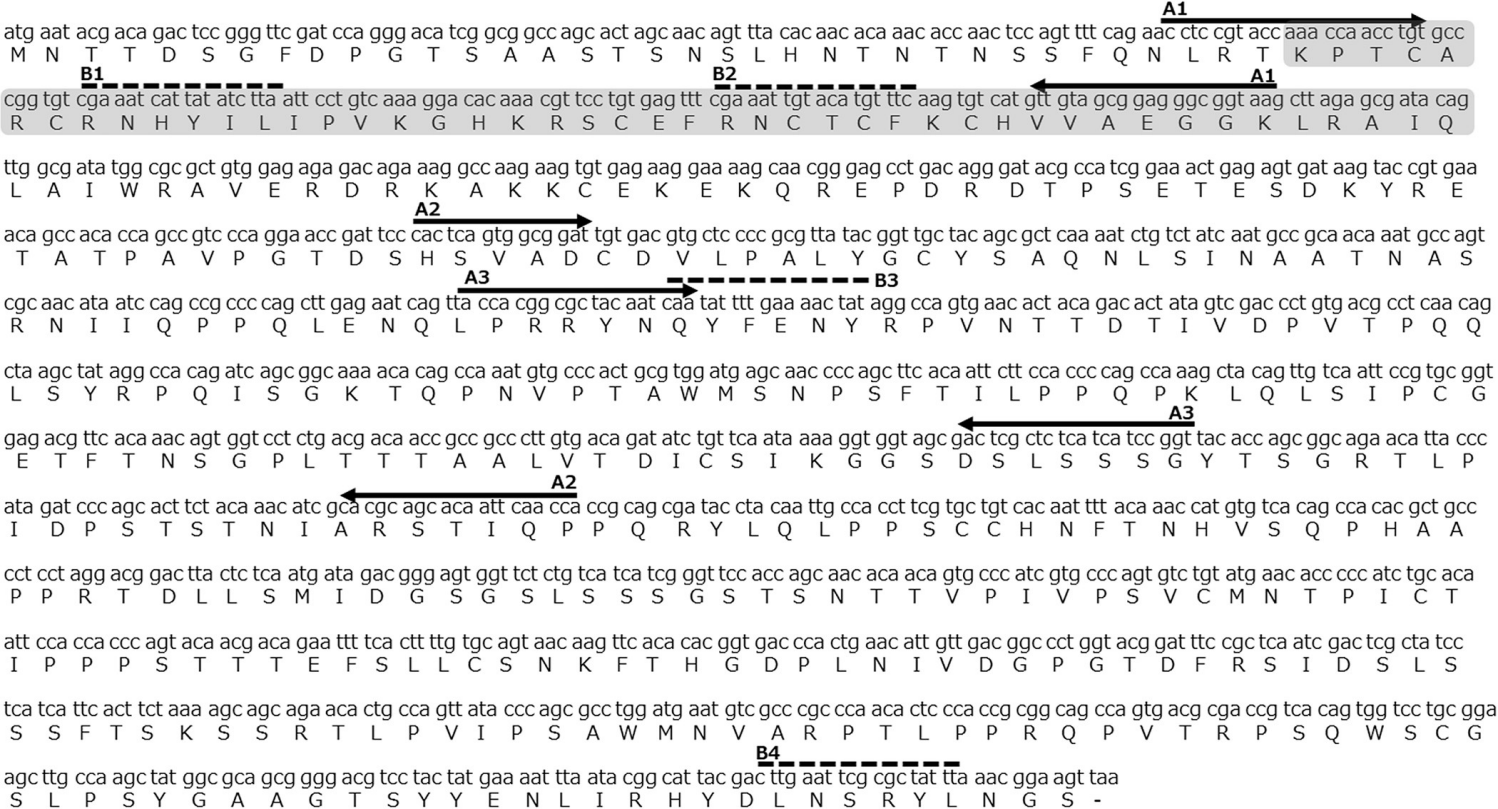
The DNA sequence of the *Doublesex* gene of *Reticulitermes speratus* (*RsDsx*, LC635717 [16]. The deduced amino acid sequences are shown under the nucleotide sequences. The conserved DM domain is indicated by the gray box. Quantitative RT-PCR was performed using a pair of primers (A1). A pair of primers (A2 and A3) were used for cloning and dsRNA synthesis, respectively. Four positions (B1-4) were used for the siRNAs targeted regions.

The most downstream gene, *Doublesex* (*Dsx*), belongs to the family of doublesex- and mab-3 related transcription factors (DMRTs) family and contains two conserved domains (doublesex/mab-3 DNA-binding domain [DM] and oligomerization domain [OD]) [8, 9]. *Dsx* is regulated by an upstream splicing regulatory factor (Transformer, Tra), and sex-specific *Dsx* isoforms are formed accordingly [10]. Male- and female-specific Dsx protein are involved in the regulation of gene expression for sex determination and sex-specific morphogenesis in holometabolous insects (e.g. [11, 12]). Two different characteristics were observed in *Dsx* genes of early branching and non-holometabolan taxa, including hemimetabolous insects. First, sex-specific *Dsx* isoforms have been constructed; however, *Dsx* is involved only in male sexual differentiation, for example, in the German cockroach *Blattella germanica* and the firebrat *Thermobia domestica* [13, 14]. Secondly, sex-specific isoforms have not been observed in some species, including the louse *Pediculus humanus*, silverleaf whitefly *Bemisia tabaci*, and termite *Reticulitermes speratus* [13, 15, 16]. In particular, the *Dsx* homolog of *R*. *speratus* (*RsDsx*) is composed of only one exon with a conserved DM domain (no OD domain) and is expressed in males but not in females (Fig 1; [16]). Termites are social cockroaches and a monophyletic group within the clade Blattodea [17–19]. *Dsx* genes of the cockroach *B*. *germanica* and *Cryptocercus punctulatus* have two conserved domains and exhibit sex-specific isoforms [13, 16]; thus, termites are one of the most important model groups for understanding *Dsx* evolution and divergence in hemimetabolous insects.

In this study, we focused on the transcriptional regulation of RsDsx target genes in the post-embryonic stage as the first step to clarify its function. The reason for targeting this species in this study is that *R*. *speratus* is (1) one of several species of termites whose genomes have been sequenced [20], (2) the first in termites in which the *dsx* structure and expression patterns among castes have been identified [16], (3) the member characterized by a bifurcated caste differentiation pathway [21], leading to morphologically distinct castes, including a reproductive caste and a non-reproductive caste comprising soldiers and workers that cannot develop into winged imagoes. According to the previous reports [12, 22], dry analysis is effective for the first step in exploring target genes of Dsx in insects other than flies. Consequently, we attempted to identify RsDsx target genes using a genome-wide analysis for binding site prediction. Based on the same criteria, we predicted Dsx target genes in three different termites with available genome information (*Reticulitermes lucifugus*, *Coptotermes formosanus*, and *Macrotermes natalensis*) (S1 Fig). Among termites with identified *Dsx*, the selected three species are positioned as follows: the species most closely related to *R*. *speratus* (*R*. *lucifugus*), the most distantly related species (*M*. *natalensis*), and a species falling in between them (*C*. *formosanus*). We examined the commonalities of candidate target genes between different species of the same genus (*R*. *lucifugus*), family (Rhinotermitidae: *C*. *formosanus*), and sister-group family (Termitidae: *M*. *natalensis*). Next, we selected several candidate target genes with significantly different expression levels between the sexes in *R*. *speratus* based on previous RNA-seq data [20]. Finally, dsRNA or siRNA injection analysis was performed in male individuals. The expression level of each candidate target gene was examined after *RsDsx* dsRNA injection. Based on the obtained results, we discuss the role of Dsx targets in termites.

## Materials and methods

### Binding motif analysis

To identify the Dsx binding motif in the four termite species, we used Hypergeometric Optimization of Motif EnRichment (HOMER) v4.11 [23]. The motif sequences used in this experiment (24 in total; S1 Table) were designed based on previous studies [24, 25]. These sequences were searched within 3.0 kb upstream from the transcription start position in each genome (RspeOGS1.0 and MnatOGS3, [20]; Rluc_AED0.5 [26]; CopFor1.0 [27]. We performed BLASTX searches against the nr database of the National Center for Biotechnology Information (NCBI) to annotate the candidate target genes of Dsx. Top-hit proteins were defined as orthologs of the candidate target genes. To obtain common target genes of Dsx between *R*. *speratus* and other termites, we utilized candidate target gene sequences from each termite species (*R*. *lucifugus*, *C*. *formosanus*, and *M*. *natalensis*) as query sequences and performed BLASTN searches against *R*. *speratus* genome data (RspeOGS1.0). The top-hit nucleotide sequences were defined as orthologs of *R*. *speratus*. The expression levels (queens and kings) of the candidate target genes in *R*. *speratus* selected using HOMER (total 101; S2 Table) were obtained from RNA-seq data [20]. Normalized RPKM data were used, and differentially expressed genes (DEGs) were selected using an FDR cutoff of 0.05. Specific primers were designed against each DEG sequence using Primer3Plus [28] (S3 Table) for real-time qPCR analysis.

### Termites

Two mature colonies of *R*. *speratus* were collected from Himi, Toyama Prefecture, Japan, in July 2019 and November 2020. Each colony was used for the collection of eggs (total 40) and last-instar N6 nymphs (500 individuals). Male and female N6 nymphs were distinguished based on the morphological characteristics of their abdominal sternites [29, 30].

### RNA extraction and cDNA synthesis

Total RNA was extracted from 40 eggs using the ReliaPrep RNA Miniprep Systems (Promega, Madison, WI, USA) or N6 nymphs (whole bodies of five individuals or abdomens of ten individuals; see below) using ISOGEN II (Nippon Gene, Tokyo, Japan). The amount of RNA and DNA was quantified using a Qubit fluorometer (Life Technology, Eugene, OR, USA), and RNA purity and quantity were measured using a Nanovue spectrophotometer (GE Healthcare BioSciences). DNase-treated RNA was used for cDNA synthesis with a high-capacity cDNA Reverse Transcription Kit (Thermo Fisher Scientific).

### Real-time quantitative PCR (qPCR) analysis

Real-time qPCR was performed using THUNDERBIRD SYBR qPCR Mix (TOYOBO, Tokyo, Japan) and a QuantStudio 3 Real-Time PCR System (Thermo Fisher Scientific) or a MiniOpticon Real-Time System (Bio-Rad, Hercules, CA, USA). According to the previous studies [16, 31], the suitability of six reference genes (i.e., *EF1-alfa* (accession no. AB602838; [32], *NADH-dh* (AB602837; [32], *beta-actin* (AB520714; [33], *Glutathione-S-transferase 1* (*GstD1*, gene ID: RS001168), *Eukaryotic initiation factor 1A* (*EIF-1*, RS005199) and *ribosomal protein S18* (*RPS18*, RS015150)) were evaluated using GeNorm [34] and NormFinder [35] software to determine an internal control gene. Specific primers were designed against each gene sequence using Primer3Plus [28] (S3 Table).

### Double-stranded RNA (dsRNA) preparation

The cDNA fragments of *RsDsx* (approximately 900 bp) were amplified from cDNA samples synthesized from egg RNA. PCR primers (position A2 in Fig 1) were designed against the gene sequence using Primer3Plus [28] (S3 Table). The PCR products were purified using a QIAquick Gel Extraction Kit (Qiagen, Tokyo, Japan) and subcloned into a pGEM-T Easy Vector (Promega, Madison, WI, USA). All constructs were sequenced using a BigDye Terminator v3.1 cycle sequencing Kit (Applied Biosystems) and an Applied Biosystems 3500 Genetic Analyzer (Applied Biosystems). Double-stranded RNA (dsRNA), including the target region, was synthesized from the plasmid DNA using gene-specific primers (position A3 in Fig 1) adapted to the T7 promoter sequences (S3 Table). In accordance with previous studies [36–39], the *GFP* sequence for the control experiment was amplified using the *GFP* vector pQBI-poll I (Wako, Osaka, Japan). *GFP*-specific primers with T7 promoter sequences have been used in previous studies [38, 39]. *RsDsx* and *GFP* dsRNAs were synthesized using T7 RNA polymerase and the MEGA Script T7 Transcription Kit (Invitrogen, Carlsbad, CA, USA).

### Small-interfering RNA (siRNA) preparation

We requested Japan Bio Services Co. LTD. (Saitama, JAPAN) to search and produce efficient and target-specific siRNA. The siRNA sequences were designed in four different regions of *RsDsx*: (guide 1, 5’-UUA AGA UAU AAU GAU UUC GAC-3’; passenger 1, 5’-CGA AAU CAU UAU AUC UUA ATT-3’; B1 in Fig 1), (guide 2, 5’-UGA AAC AUG UAC AAU UUC GAA-3’; passenger 2, 5’-CGA AAU UGU ACA UGU UUC ATT-3’; B2 in Fig 1), (guide 3, 5’-UAU AGU UUU CAA AAU AUU GAT-3’; passenger 3, 5’-CAA UAU UUU GAA AAC UAU ATT-3’; B3 in Fig 1), (guide 4, 5’-UAA AUA GCG CGA AUU CAA GTC-3’; passenger 4, 5’-CUU GAA UUC GCG CUA UUU ATT-3’; B4 in Fig 1). The *GFP* siRNA sequences for control experiment were as follows: (guide, 5’-GUU GUA GUU GUA CUC CAG CTT-3’; passenger, 5’-GCU GGA GUA CAA CUA CAA CTT-3’). Following the protocol provided by Japan Bio Services Co. LTD, the guide and passenger siRNA were mixed with annealing buffer. The solution was heated at 90°C for 1 min, and stand at 37°C for 60 min. Four *RsDsx* siRNAs were combined in equal amounts (final concentration: 20 pmol/μL).

### dsRNA injection

Because the expression levels of *RsDsx* were relatively low (RPKM values below 4; [16], many individuals should be used for gene expression analysis. Although *RsDsx* is highly expressed in kings [16], securing a large number of individuals is challenging. Consequently, we used the last instar (sixth stage) nymphs (N6; [40] collected from the field colony. Male N6 nymphs were anesthetized on ice for 90 seconds, and dsRNA solution (2.5 μg in the 202.4 nL nuclease-free water) was injected into the thorax using a Nanoliter 2000 (World Precision Instruments, Sarasota, FL, USA) in accordance with the previous method [37, 39]. The dsRNA-injected N6 nymphs were maintained in 46 mm Petri dishes with moistened filter paper (15 individuals per dish). All dishes were incubated at 25°C in constant darkness. The N6 nymphs were collected three and six days after dsRNA injection (n = 75 individuals per day) to evaluate the change in the expression levels of *RsDsx* and several other genes. Different expression levels of *RsDsx* and *GFP* dsRNA-injected N6 nymphs were examined using real-time qPCR analysis. Because the knockdown effects of *RsDsx* were not observed (see Results), dsRNA injection analysis was performed again, and dsRNA-injected N6 nymphs were collected one day after dsRNA injection (n = 75 individuals). Total RNA was extracted from the whole bodies of five nymphs from each sample (biological replications = 15) (S2 Fig). Statistical analysis was performed using the Mann–Whitney U test with Mac Statistical Analysis ver. 2.0 (Esumi, Tokyo, Japan).

### siRNA injection

Male N6 nymphs were anesthetized on ice for 90 seconds, and siRNA solution (202.4 nL) was injected into the thorax using a Nanoliter 2000. The siRNA-injected N6 nymphs were maintained using the same method described above. The N6 nymphs were collected one and three days after siRNA injection (n = 45 individuals per day) to evaluate the change in the expression levels of *RsDsx*. Different expression levels of *RsDsx* and *GFP* siRNA-injected N6 nymphs were examined using real-time qPCR analysis. Total RNA was extracted from the whole bodies of five nymphs from each sample (biological replications = 9). Statistical analysis was performed using the same method described above.

## Results and discussion

### Candidate target genes of Dsx in termites

**Fig 2:**
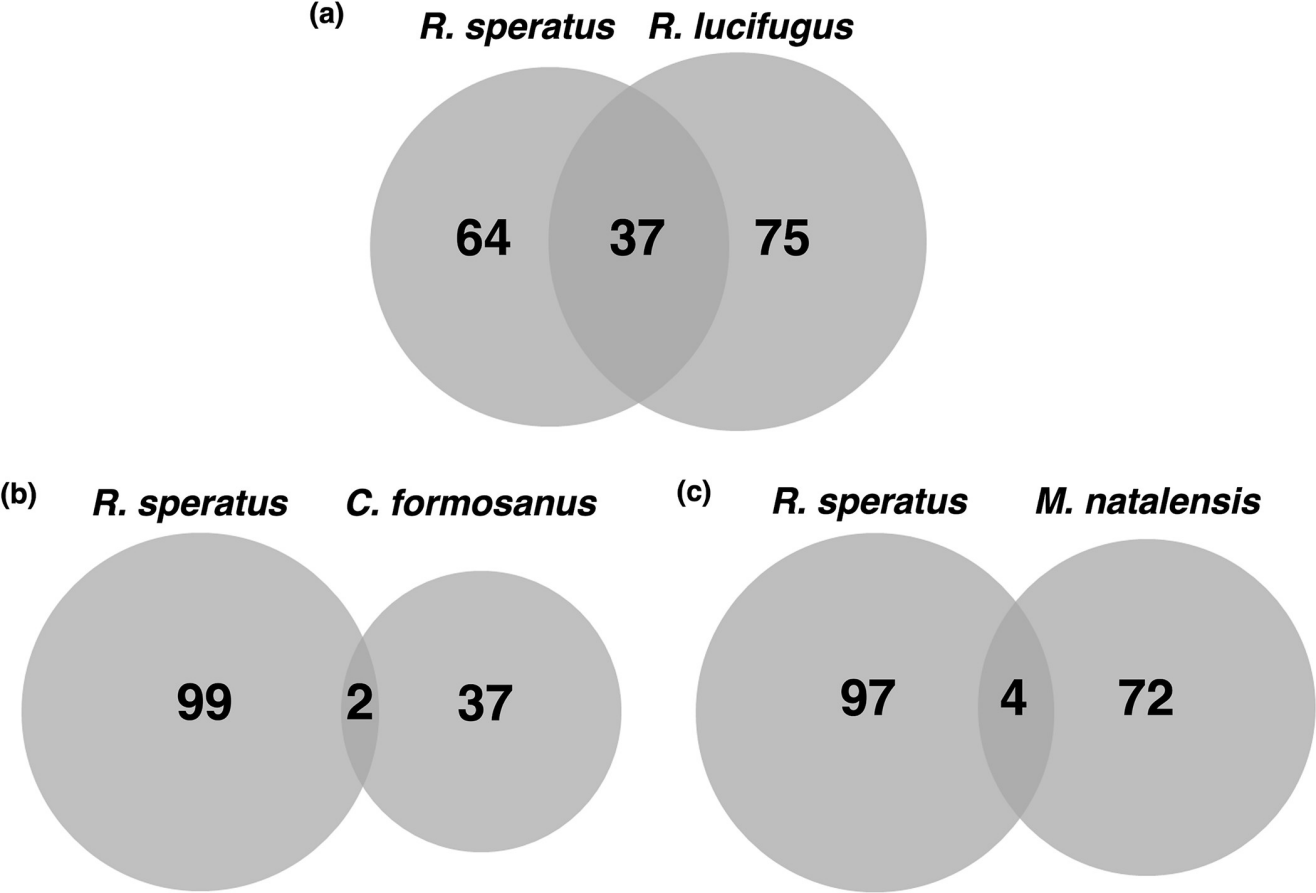
Comparison of target genes between termite species. Venn diagrams showing the numbers of candidate Dsx target genes of *R*. *speratus* and *R*. *lucifugus* (a), *R*. *speratus* and *C*. *formosanus* (b), and *R*. *speratus* and *M*. *natalensis* (c).

To obtain the downstream candidate target genes of RsDsx (Fig 1, [16]), we searched Dsx binding motif sequences in the *R*. *speratu*s genome [20] using HOMER v4.11. We identified 101 candidate target genes of RsDsx with motif sequences within 3.0 kb upstream of the transcription start position (S2 Table). Using the same method, we searched for candidate target genes of Dsx in three other termite species (*R*. *lucifugus*, *C*. *formosanus*, and *M*. *natalensis*). We identified 112 candidate target genes in *R*. *lucifugus* (S4 Table), 39 genes in *C*. *formosanus* (S5 Table), and 76 genes in *M*. *natalensis* (S6 Table). In the comparison between *R*. *speratus* and *R*. *lucifugus*, we found that 37 genes were common candidate Dsx targets in both species (Fig 2A). However, when we compared the candidate genes between *R*. *speratus* and *C*. *formosanus* or *M*. *natalensis*, we found only a few common target genes in two species (two or four genes, respectively; Fig 2B and 2C) and no common targets across all four species. These results suggest that Dsx target genes are less conserved and that downstream Dsx regulation is diverse in termites.

Out of 37 candidate target genes shared between two *Reticulitermes* species (refer to S2 and S4 Tables), at least eight genes have been proposed to play a role in male spermatogenesis (Table 1). In various insect lineages, the knockdown or knockout of *Dsx* resulted in the incomplete development of reproductive organs [12–14, 41–44]. We suggest that these eight genes are directly regulated by Dsx and involved in the regulation of spermatogenesis in *Reticulitermes*. At least four genes have been suggested to be involved in the longevity of some animals, including nematodes (Table 1). In eusocial insects, reproductives normally live longer than sterile castes, sometimes even for decades [45]. In addition, in some termite species, including *R*. *speratus* and *R*. *lucifugus*, which have an asexual queen succession (AQS) system, the primary king is not replaced and is normally maintained in the colony [46–48]. In species with the AQS, the primary king may live longer than the primary queen [46]. These longevity-related genes were not included as candidate targets in *C*. *formosanus* or *M*. *natalensis*, both of which lack an AQS system. Further analyses on the targets of *Dsx* should be performed in termites with or without the AQS.

**Table 1:** List of RsDsx target genes probably involved in spermatogenensis and longevity.

Gene ID of *R*. *speratus*	Gene ID of *R*. *lucifugus*	Blastx (nr) top hit [species]^1^	Probable function	Taxon (References)
RS001786	RlucM00000015181	E3 ubiquitin-protein ligase SIAH1A [*Cryptotermes secundus*]	Spermatogenesis	Some animals [49, 50]
RS001881	RlucM00000001135	RNA-binding protein 42-like [*Zootermopsis nevadensis*]		Mouse [51]
RS005429	RlucM00000001468	engulfment and cell motility protein 1 [*Cryptotermes secundus*]		Mouse [52]; *Caenorhabditis elegans* [53]
RS009093	RlucM00000009988	myosin-1 [*Zootermopsis nevadensis*]		Some mammals [54, 55]
RS011008	RlucM00000006817	piwi-like protein Siwi isoform X2 [*Zootermopsis nevadensis*]		*Bombyx mori* [56, 57]
RS013339	RlucM00000009648	Actin-related protein 3 [*Blattella germanica*]		Some mammals [54]
RS014159	RlucM00000002478	cyclin-K isoform X3 [*Zootermopsis nevadensis*]		*Bactrocera dorsalis* [58]
RS014714	RlucM00000015295	E3 ubiquitin-protein ligase SIAH1A [*Cryptotermes secundus*]		Some animals [49, 50]
RS000654	RlucM00000003372	transforming growth factor beta regulator 1 isoform X2 [*Cryptotermes secundus*]	Longevity	*C*. *elegans* [59]
RS006416	RlucM00000007367	SH2B adapter protein 1 isoform X1 [*Cryptotermes secundus*]		*Drosophila* [60]
RS009246	RlucM00000010834	B-box type zinc finger protein ncl-1 isoform X1 [*Cryptotermes secundus*]		*C*. *elegans* [61]
RS014227	RlucM00000000860	40S ribosomal protein S6 [*Cryptotermes secundus*]		*C*. *elegans* [62]; eukaryotes [63]

^1^Accessed on 9 November 2023.

### Selected candidate target genes of RsDsx

Among the candidate genes obtained from *R*. *speratus* (total 101), we selected genes with significantly different expression levels between female and male reproductives (queens and kings) using RNA-seq data (head and thorax + abdomen samples; [20]). Nine genes were differentially expressed between the sexes (FDR < 0.05) (S2 Table). Among these, six and three genes were highly expressed in kings and queens, respectively (Table 2). King-biased genes may be promoted in transcription by Dsx. Especially, two out of the six king-biased genes (*piwi-like protein* (*piwi*) and *B-box type zinc finger protein* (*B-box*)) were identified as common candidate target genes in both *R*. *speratus* and *R*. *lucifugus* (Table 1). The *piwi* gene plays an important role in silkworm spermatogenesis [56, 57], while the *B-box* gene is involved in regulating the lifespan of the nematode *Caenorhabditis elegans* [61]. While the presence of queen-biased genes (*Vitellogenin* (*Vg*), *Beta-glucosidase*, and *Proteoglycan4*) suggests that *RsDsx*, expressed only in males [16], functions not only as a transcriptional activator but also as a repressor in the regulation of female-biased genes in males. In *Drosophila melanogaster*, Dsx has been shown to function as both an activator of *Yolk proteins* and *bric-a-brac* in females and a repressor of those in males [64]. We propose that *piwi* and *B-box* genes are important targets for determining whether *Dsx* is involved in the development of male-specific termite traits.

**Table 2:** RsDsx target genes with sex-specific expression patterns.

Gene ID	Higher expression levels in RNA-seq data^1^	Motif sequences	Blastx (nr) top hit [species]^2^
RS000610	Queen	ATAACAATGTTAT	vitellogenin-1-like isoform X1 [*Zootermopsis nevadensis*]
RS004624	Queen	GAAACTAAGTTTC	beta-glucosidase [*Coptotermes formosanus*]
RS005447	King	AAAACAATGTTTT	cilia- and flagella-associated protein 44 [*Cryptotermes secundus*]
RS006266	King	AATACTAAGTATT	hypothetical protein Cfor_01712 [*Coptotermes formosanus*]
RS007010	Queen	GCAACAATGTTGC	hypothetical protein Cfor_03964 [*Coptotermes formosanus*]
RS009246	King	AAAACAATGTTTT	B-box type zinc finger protein ncl-1 isoform X1 [*Cryptotermes secundus*]
RS009322	King	ATTACTAAGTAAT	hypothetical protein Cfor_12321 [*Coptotermes formosanus*]
RS011008	King	AATACTAAGTATT	piwi-like protein Siwi isoform X2 [*Zootermopsis nevadensis*]
RS014546	King	GCAACAATGTTGC	transient receptor potential cation channel protein painless-like [*Zootermopsis nevadensis*]

^1^Expression differences between queens and kings were examined using RNA-seq data (Shigenobu et al. 2022).

^2^Accessed on 9 November 2023.

### Expression levels of *RsDsx* after dsRNA/siRNA injection

**Fig 3:**
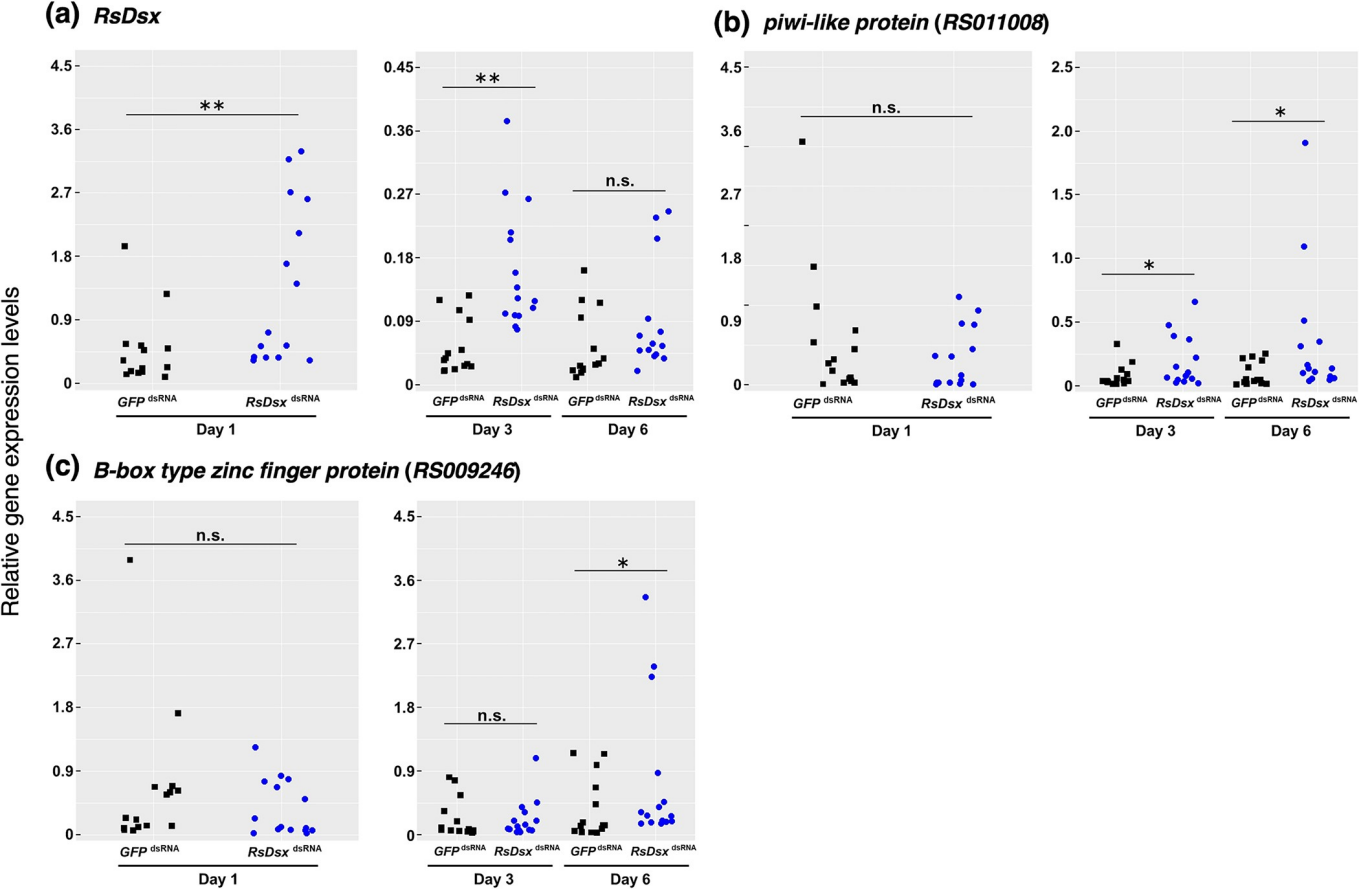
*RsDsx* dsRNA injection affects the expression of genes associated with spermatogenesis and lifespan regulation. Quantitative real-time PCR expression analysis in dsRNA-injected male nymphs of *R*. *speratus*. Expression levels (number of biological replications = 15) of *RsDsx* (a), *piwi-like protein* (b), and *B-box type zinc finger protein* (c) were compared between *GFP* and *RsDsx* dsRNA-injected individuals one, three, and six days after the injection. Asterisks indicate significant differences (Mann–Whitney U test, P < 0.01**, P < 0.05*). n.s. means no significant differences.

Both GeNorm and NormFinder software showed that *beta-actin* (dsRNA injection) or *GstD1* (siRNA injection) expression levels were the most stable (S7 Table). First, we performed real-time qPCR using N6 nymphs three and six days after dsRNA injection and then performed qPCR analysis using individuals just one day after the injection. Surprisingly, *RsDsx* expression levels were significantly increased by *RsDsx* dsRNA injection compared to *GFP* dsRNA injection one and three days after treatment (Fig 3A). Similarly, *RsDsx* expression levels were significantly increased by *RsDsx* siRNA injection compared to the control (S3 Fig). However, these results consistent with those obtained in a previous analysis of the cockroach *B*. *germanica* [13]. RNAi of *B*. *germanica Dsx* (*BgDsx*) resulted in increased *BgDsx* expression levels compared to the *GFP* control in male nymphs both six hours and three days after dsRNA treatment. As suggested by Wexler et al. (2019), this is probably due to the rebound effect of targeted gene expression and transcription. Alternatively, there is a possibility that lineage-(cockroaches and termites) specific problems are caused by *Dsx* RNAi because expression of *Tra* (upstream gene of *Dsx* in many insects) was successfully decreased by RNAi in *B*. *germanica* [13]. However, the proximal mechanism by which *Dsx* expression increases with *Dsx* dsRNA injection is unclear in both *R*. *speratus* and *B*. *germanica*. Further analysis is required to clarify this issue.

### *RsDsx* dsRNA injection effects on the expression of candidate target genes

When the expression levels of *Dsx* vary, genes with Dsx binding sites that exhibit concurrent expression changes are considered to be target genes of Dsx. Then we performed expression analysis of candidate target genes after *RsDsx* dsRNA injection. We performed real-time qPCR analysis of the target genes (Table 2) in dsRNA-injected N6 nymphs one, three, and six days after dsRNA injection. We focused on two common candidate target genes of *R*. *speratus* and *R*. *lucifugus* (*RS011008* and *RS009246*; Table 1). The expression levels of *piwi-like protein* (*RS011008*) were significantly increased in the *RsDsx* dsRNA injection treatments compared to those in the *GFP* controls three and six days after dsRNA injection (Fig 3B). High *piwi-like* expression levels might be facilitated by the upregulation of *RsDsx* at one and three days after treatment. Similarly, the expression of *B-box type zinc finger protein* (*RS009246*) was significantly increased in *RsDsx* dsRNA-injected nymphs six days after injection (Fig 3C). To provide an additional evidence of gene expression regulation by RsDsx, we examined if there was a correlation between the expression levels of *RsDsx* and its potential target genes. The results indicated that a significant correlation was observed both in *RsDsx* and *GFP* dsRNA-injected nymphs (S4 Fig). These results also indicate that both genes might be under the control of RsDsx. Furthermore, we observed the expression levels of two house-keeping genes (*NADH-dh* and *GstD1*) after dsRNA injection. Expression levels of both genes were not affected by *RsDsx* dsRNA injection (S5 Fig). Consequently, the non-specific effects due to dsRNA injection might not be generally occurring. Previous study showed that *Dsx* was highly expressed in kings compared to other castes [16]. Termite kings are long-lived, possessing well-developed testes, and maintaining a high reproductive capacity. The current findings suggest that the observed target genes are associated with these characteristics of termite kings.

We could not quantify the expression levels of one gene (*RS014546*) because its expression levels were too low. The expression levels of the remaining six genes were not significantly different between *RsDsx* and *GFP* dsRNA injection treatments, except for *Vg* (*RS000610*), which increased in *RsDsx*-treated nymphs three days after the injection (S6 Fig). *Vg* is a precursor of the major yolk protein and is crucial for female vitellogenesis in most insects [65]. Four homologous *Vg* sequences have been found in *R*. *speratus* [66], and the *Vg* gene (*RS000610*; called *RsVgI*) is highly expressed in female reproductives [33, 37, 66]. Expression of *Vg* was also increased by *BgDsx* RNAi treatment in *B*. *germanica* male individuals [13]. In contrast, expression levels of *Vg* were suppressed by male-specific *Dsx* isoforms in some insect species [13–15, 67–69]. In the case of *B*. *germanica*, an increase in *BgDsx* expression was observed following *BgDsx* dsRNA injection. However, this result is thought to be influenced by the rebound effect [13]. Therefore, the subsequent increase in *Vg* expression after *BgDsx* dsRNA injection is presumed to be a result of the lowered expression level of *BgDsx*, leading to the release of its repressor function on *Vg*. While a similar possibility exists in termites, the precise molecular relationships remain unclear, because the knockdown of *RsDsx* could not be confirmed in *R*. *speratus* (Fig 3A). In addition, *Vg* was not included among the candidate *Dsx* target genes in the other termites examined (*R*. *lucifugus*, *C*. *formosanus*, and *M*. *natalensis*; S4–S6 Tables). Further functional analyses are required to determine whether male-specific *Dsx* is involved in the regulation of *Vg* expression in termites.

## Conclusions

We examined *Dsx* target motifs in the genome sequences of the four termite species. There were 37 common candidate target genes between *R*. *speratus* and *R*. *lucifugus*; however, few were identified in the comparison between *R*. *speratus* and *C*. *formosanus/M*. *natalensis* and none were found across all four species. Based on the RNA-seq data and the results of *RsDsx* dsRNA injection, we identified at least two candidate target genes in *R*. *speratus*, both of which were highly expressed in males and included in the candidate targets observed in *R*. *lucifugus*. In termites, unlike ants and bees, kings continue to exist within the colony while maintaining their reproductive capabilities. The pivotal role of *Dsx*, expressed exclusively in males, in influencing the expression of genes associated with spermatogenesis and lifespan in kings is deemed essential for the proper maintenance of termite social structure. Furthermore, the unique structure of termite *Dsx* and its male-specific expression pattern offer the potential for applications in pest management and biological control, such as the development of species-specific interfering RNA pesticides. Information observed in this study will be useful for future analyses of the *Dsx* role in termites, which exhibit a distinctive *Dsx* structure and unique expression pattern among insects.

## Supporting information

S1 Fig Phylogenetic relationships of termites with genome information and related cockroaches. (TIFF)

S2 Fig Diagram of dsRNA injection analysis. Experimental design for dsRNA injection analysis. Male last instar nymphs (N6 stage) were obtained from a mature colony, and dsRNA injection was performed in each individual. All dsRNA-injected N6 nymphs were maintained in Petri dishes (15 individuals/dish). Total RNA was extracted from whole bodies of five individuals, and the biological replications of extracted RNA (n = 15) were prepared.
(TIFF)

S3 Fig Expression analysis of *RsDsx* after siRNA injection. Quantitative real-time PCR expression analysis in siRNA-injected male nymphs of *R*. *speratus*. Expression levels (number of biological replications = 9) of *RsDsx* were compared between *GFP* and *RsDsx* siRNA-injected individuals one and three days after the injection. Asterisks indicate significant differences (Mann–Whitney U test, P < 0.05*). n.s. means no significant differences.
(TIFF)

S4 Fig A correlation between the expression levels of *RsDsx* and its potential target genes. Expression levels of *RsDsx* (x-axis) and *piwi-like protein* (y-axis) in each dsRNA-injected nymph (a, b) and *B-box type zinc finger protein* (y-axis) in each dsRNA-injected nymph (c, d). The dashed line represents the correlation line, and R^2^ indicates the square of the correlation coefficient.
(TIFF)

S5 Fig Expression analysis of *NADH-dh* and *GstD1* after dsRNA injection. Quantitative real-time PCR expression analysis in dsRNA-injected male nymphs of *R*. *speratus*. Expression levels (number of biological replications = 14) of *NADH-dh* (a) and *GstD1* (b) were compared between *GFP* and *RsDsx* dsRNA-injected individuals three days after the injection. n.s. means no significant differences (Mann–Whitney U test, P < 0.05).
(TIFF)

S6 Fig Expression analysis of other candidate target genes after *RsDsx* dsRNA injection. Quantitative real-time PCR expression analysis in dsRNA-injected male nymphs of *R*. *speratus*. Expression levels (number of biological replications = 13–15) of *vitellogenin* (a), *cilia- and flagella-associated protein* (b), *proteoglycan 4-like* (c), *hypothetical protein* (*RS006266*) (d), *hypothetical protein* (*RS009322*) (e), and *beta-glucosidase* (f) were compared between *GFP* and *RsDsx* dsRNA-injected individuals three and six days after the injection. The numerals in parentheses represent the number of biological replications. Asterisks indicate significant differences (Mann–Whitney U test, P < 0.05*). n.s. means no significant differences.
(TIFF)

S1 Table Dsx binding motif sequences used in this study. (XLSX)

S2 Table Target genes of RsDsx identified by HOMER v4.11 and expression differences between queens and kings. (XLSX)

S3 Table Primer sequences used in this study. (XLSX)

S4 Table Target genes of *Reticulitermes lucifugus* Dsx (RlDsx) identified by HOMER v4.11. (XLSX)

S5 Table Target genes of *Coptotermes formosanus* Dsx (CfDsx) identified by HOMER v4.11. (XLSX)

S6 Table Target genes of *Macrotermes natalensis* Dsx (MnDsx) identified by HOMER v4.11. (XLSX)

S7 Table Stability values of reference genes in real-time qPCR analysis after dsRNA injection. (XLSX)
