# Supplementary figures and images for: Candidate target genes of the male-specific expressed Doublesex in the termite Reticulitermes speratus
Source: PLoS One. 2024 Mar 1;19(3):e0299900. doi: 10.1371/journal.pone.0299900 (PMC10906832; doi:10.1371/journal.pone.0299900)

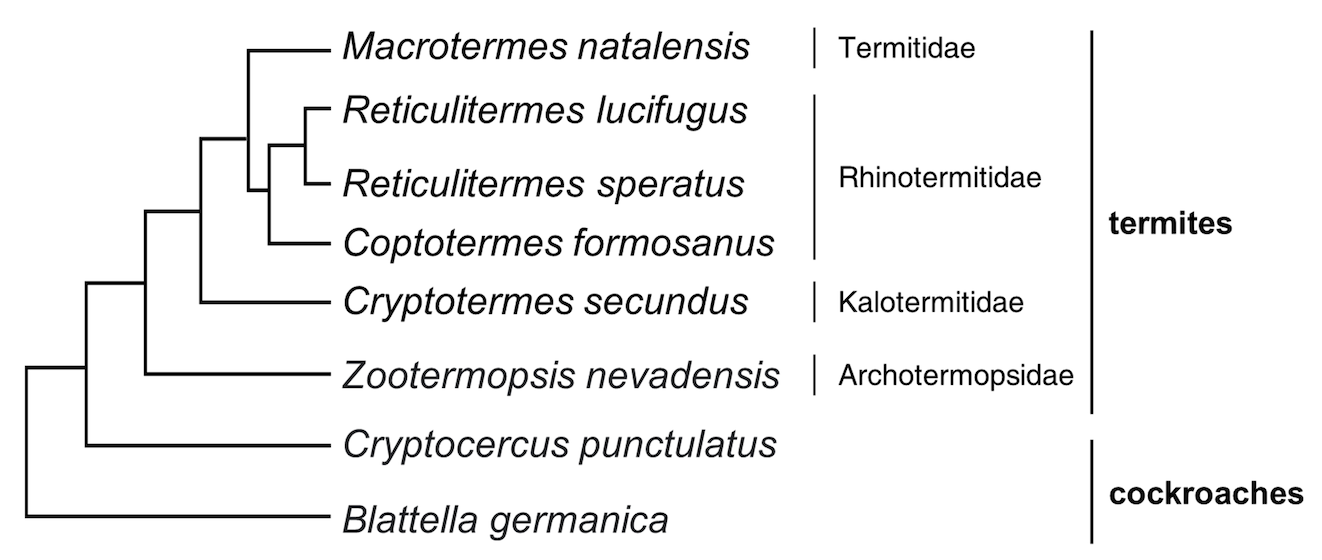

Supplement: S1 Fig — (TIFF) [file pone.0299900.s001.tiff]

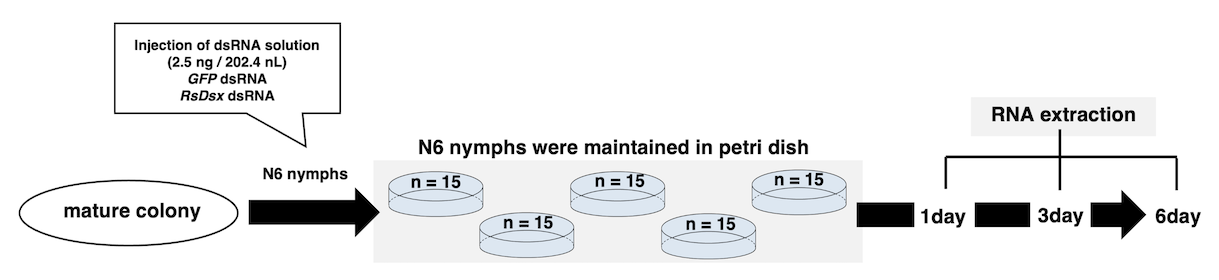

Supplement: S2 Fig — Experimental design for dsRNA injection analysis. Male last instar nymphs (N6 stage) were obtained from a mature colony, and dsRNA injection was performed in each individual. All dsRNA-injected N6 nymphs were maintained in Petri dishes (15 individuals/dish). Total RNA was extracted from whole bodies of five individuals, and the biological replications of extracted RNA (n = 15) were prepared. (TIFF) [file pone.0299900.s002.tiff]

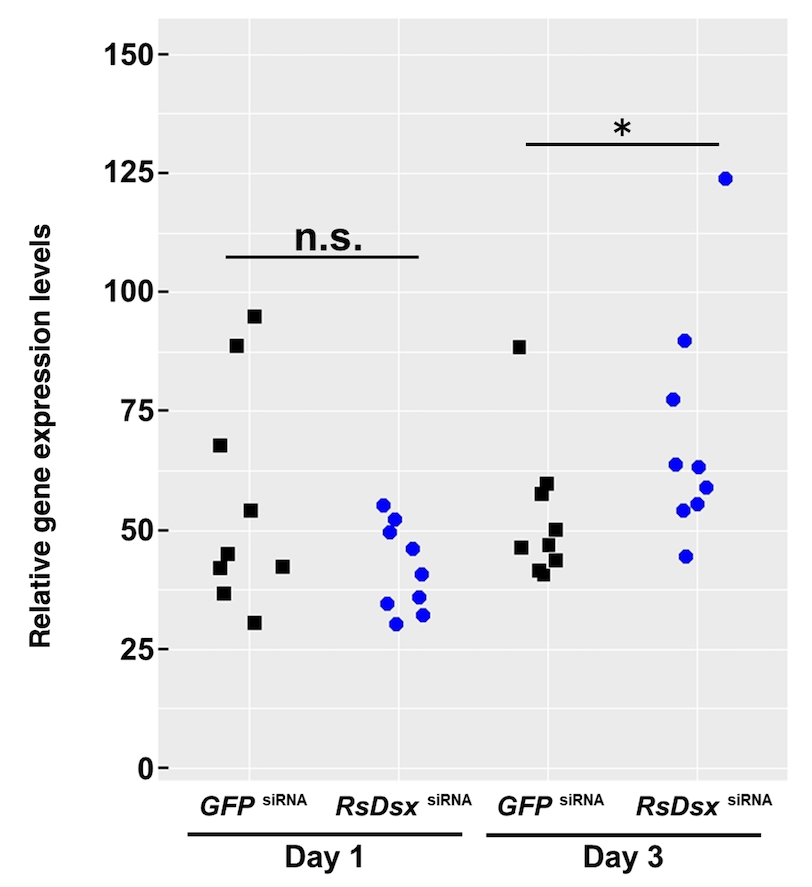

Supplement: S3 Fig — Quantitative real-time PCR expression analysis in siRNA-injected male nymphs of R. speratus. Expression levels (number of biological replications = 9) of RsDsx were compared between GFP and RsDsx siRNA-injected individuals one and three days after the injection. Asterisks indicate significant differences (Mann–Whitney U test, P < 0.05*). n.s. means no significant differences. (TIFF) [file pone.0299900.s003.tiff]

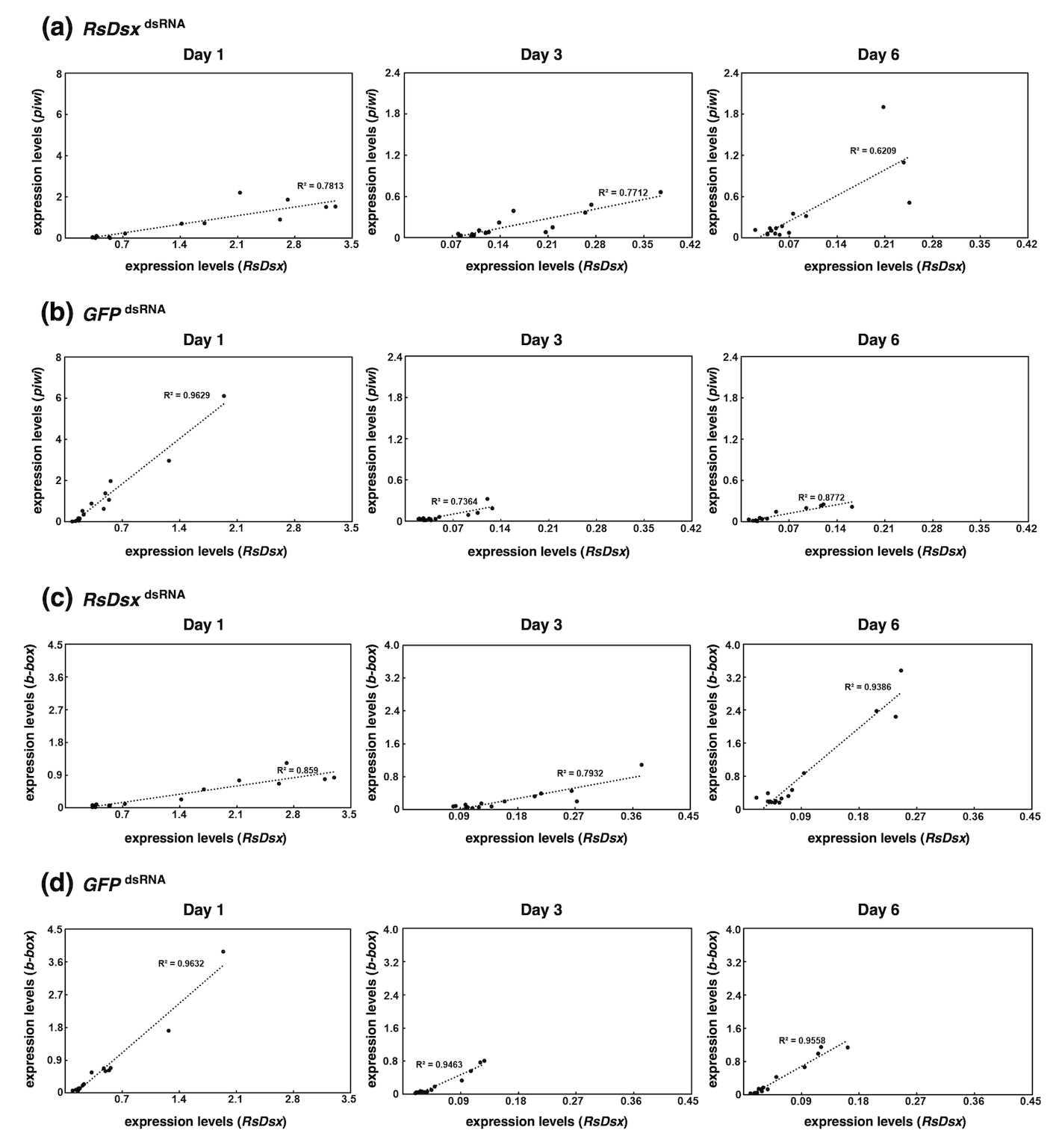

Supplement: S4 Fig — Expression levels of RsDsx (x-axis) and piwi-like protein (y-axis) in each dsRNA-injected nymph (a, b) and B-box type zinc finger protein (y-axis) in each dsRNA-injected nymph (c, d). The dashed line represents the correlation line, and R2 indicates the square of the correlation coefficient. (TIFF) [file pone.0299900.s004.tiff]

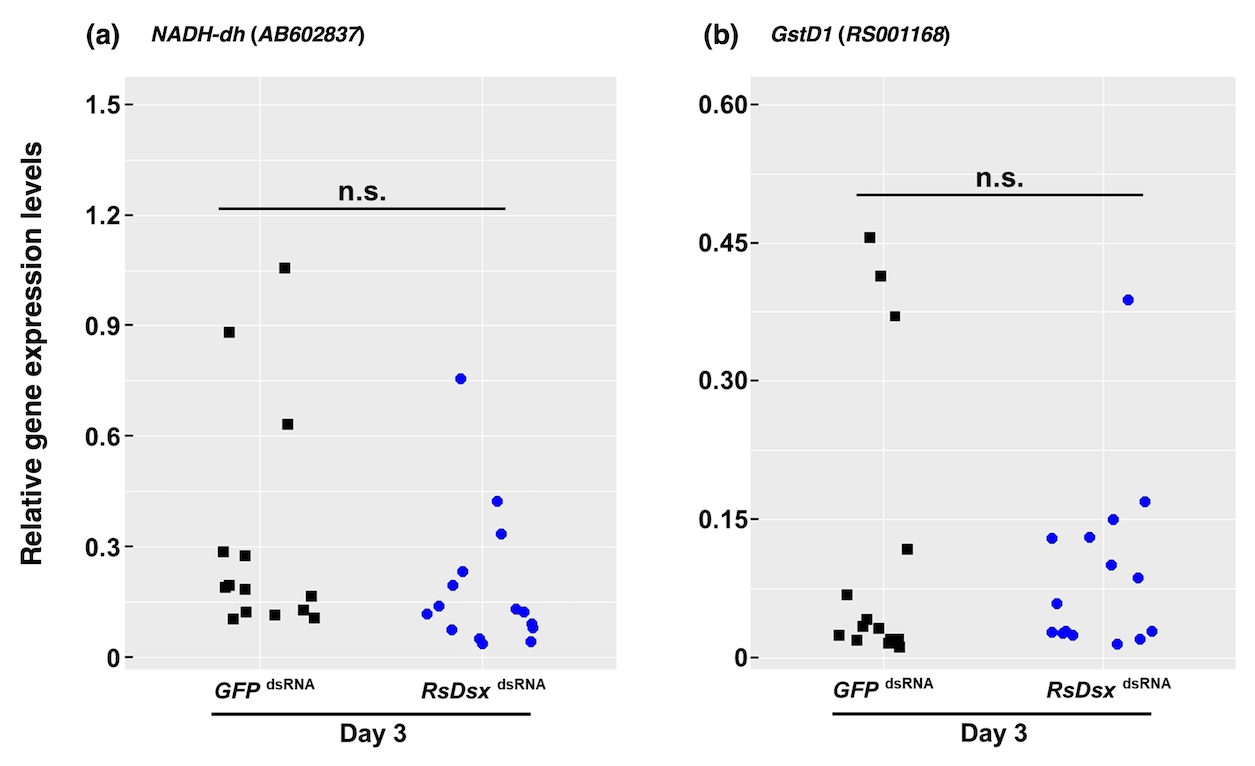

Supplement: S5 Fig — Quantitative real-time PCR expression analysis in dsRNA-injected male nymphs of R. speratus. Expression levels (number of biological replications = 14) of NADH-dh (a) and GstD1 (b) were compared between GFP and RsDsx dsRNA-injected individuals three days after the injection. n.s. means no significant differences (Mann–Whitney U test, P < 0.05). (TIFF) [file pone.0299900.s005.tiff]

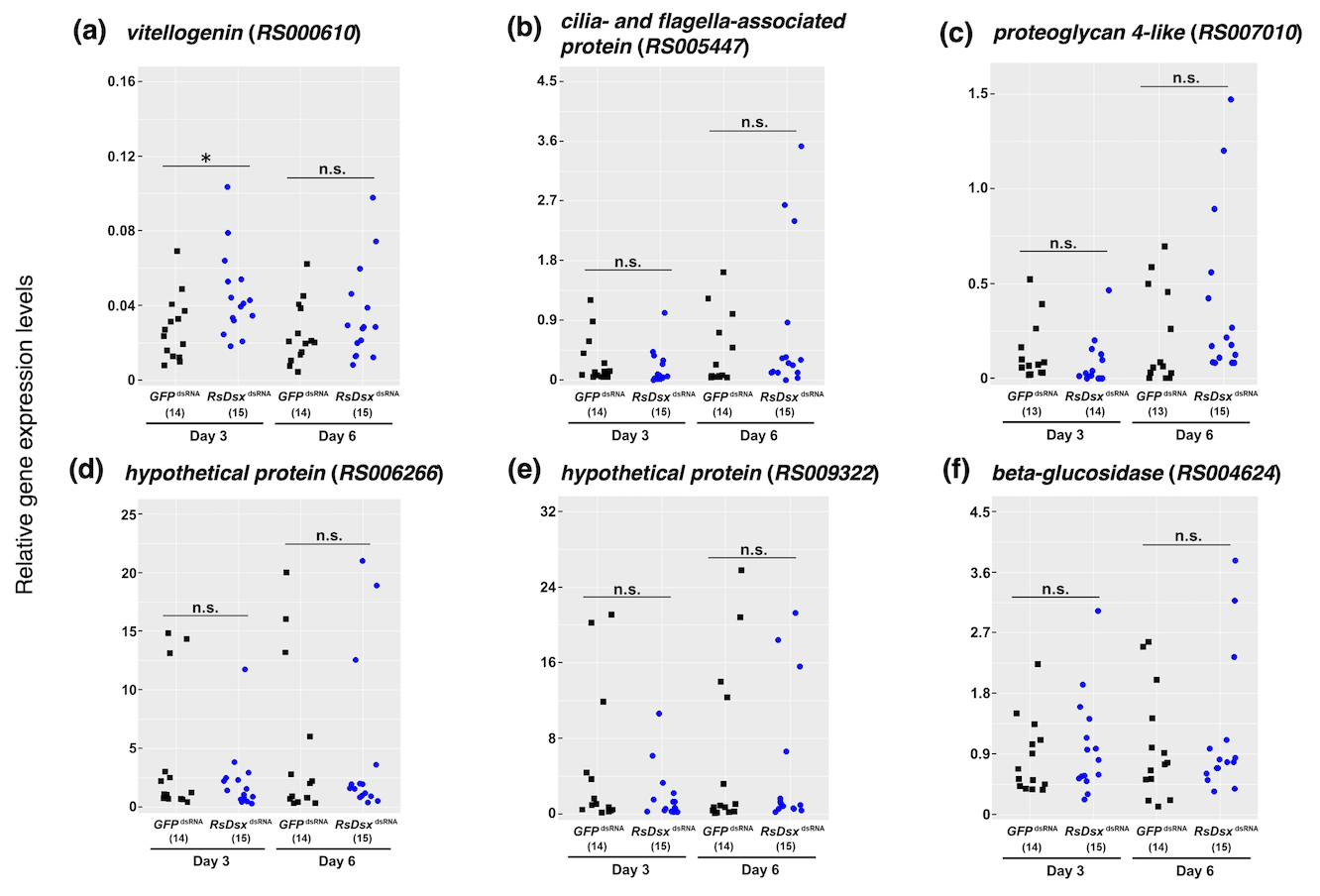

Supplement: S6 Fig — Quantitative real-time PCR expression analysis in dsRNA-injected male nymphs of R. speratus. Expression levels (number of biological replications = 13–15) of vitellogenin (a), cilia- and flagella-associated protein (b), proteoglycan 4-like (c), hypothetical protein (RS006266) (d), hypothetical protein (RS009322) (e), and beta-glucosidase (f) were compared between GFP and RsDsx dsRNA-injected individuals three and six days after the injection. The numerals in parentheses represent the number of biological replications. Asterisks indicate significant differences (Mann–Whitney U test, P < 0.05*). n.s. means no significant differences. (TIFF) [file pone.0299900.s006.tiff]
